# The Inhibition of Wnt Restrain KRAS^G12V^-Driven Metastasis in Non-Small-Cell Lung Cancer

**DOI:** 10.3390/cancers12040837

**Published:** 2020-03-31

**Authors:** Pei-Shan Hung, Ming-Hung Huang, Yuan-Yeh Kuo, James Chih-Hsin Yang

**Affiliations:** 1 Graduate Institute of Oncology, College of Medicine, National Taiwan University, Taipei 100, Taiwan; orange366@hotmail.com (P.-S.H.); mh122866@gmail.com (M.-H.H.); 2 Tai-Chen Cell Therapy Center, National Taiwan University, Taipei 100, Taiwan; d90442001@ntu.edu.tw; 3 Department of Oncology, National Taiwan University Hospital, Taipei 100, Taiwan; 4 National Taiwan University Cancer Center, College of Medicine, National Taiwan University, Taipei 100, Taiwan

**Keywords:** metastasis, NSCLC, *KRAS^G12D^*, *KRAS^G12V^*, Wnt/β-catenin, RhoA

## Abstract

The *KRAS* mutations have been an obstacle to identify therapeutic targets in cancer treatment. In this work, we clarified the distinct metastasis pattern of non-small-cell lung carcinoma (NSCLC) induced by KRAS^G12V^/KRAS^G12D^ mutations and inhibited the KRAS^G12V^ mediated metastasis by Wnt inhibitor. First, we found that KRAS^G12V^ induced more aggressive phenotype in vitro and in vivo experiments. The Gene Set Enrichment Analysis (GSEA) results of H838 KRAS^G12V^ cells showed a significant negative correlation with RhoA-related signaling. Following this clue, we observed KRAS^G12D^ induced higher activation of RhoA and suppressed activation of Wnt/β-catenin in H838KRAS^G12D^ cells. The restored activation of Wnt/β-catenin in H838KRAS^G12D^ cells could be detected when expression with a dominant-negative mutant of RhoA or treatment with RhoA inhibitor. Furthermore, the Wnt inhibitor abolished the KRAS^G12V^-induced migration. We elucidated the importance of the axis of RhoA/Wnt in regulatory NSCLC metastasis driven by *KRAS* mutations. Our data indicate that KRAS^G12V^ driven NSCLC metastasis is Wnt-dependent and the mechanisms of NSCLC metastasis induced by KRAS^G12V^/KRAS^G12D^ is distinct.

## 1. Introduction

KRAS mutations occur in approximately 30% of all human cancers, notably occurring in the most lethal cancers such as pancreatic cancer, colon cancer and lung cancer. Ras as a most frequently mutated oncogene and associated with particular poor disease prognoses in cancer, the enormous effort had been made to develop drugs that target KRAS mutations directly or indirectly. Several of the candidate drugs that inhibit KRAS^G12C^ mutation directly were developed and showed treatment efficacy both in vitro and in vivo [1,2]. The Ras protein regulate signaling pathways crucial for cell growth, migration, adhesion, cytoskeletal integrity, survival and differentiation [3]. Under normal physiological conditions, the activity of RAS proteins is tightly regulated [4,5]. The missense mutations of KRAS are frequently found in codons G12, G13 and Q61, and the missense mutations cause KRAS-guanosine triphosphate (GTP) to become locked in an activated state and persist in activating Ras signaling [6,7,8,9]. The KRAS-mutant lung cancer are more common in Western population (26%), but in Asia population only 11%. The KRAS^G12C^ and KRAS^G12V^ mutations are predominant in smokers, while the KRAS^G12D^, KRAS^G12S^ and KRAS^G13D^ mutations occur more frequently in never smokers [10,11,12].

Currently, much evidence has noted that not all KRAS mutations are created equal [13]. The advanced non-small-cell lung carcinoma (NSCLC) patients with KRAS^G12V^ mutation had a significantly shorter progression-free survival (PFS) than patients with KRAS^WT^ or non-KRAS^G12V^ mutation [14]. The recurrence sites of lung cancer patients with KRAS^G12C^ mutation were predictive of bone metastasis, but patients with KRAS^G12V^ mutation were predictive of pleuro-pericardial metastasis. In addition, the lung cancer patients with KRAS^G12V^ exhibited worse overall survival (OS) and higher recurrence incidences [15,16]. The lung cancer patients with different KRAS mutations display distinct sensitivity to chemotherapy and targeted therapy [17,18,19]. In the Biomarker-integrated Approaches of Targeted Therapy for Lung Cancer Elimination (BATTLE) trial, Ihle, N.T. et al. reported that lung adenocarcinoma patients with KRAS^G12C/^KRAS^G12V^ mutations had significantly poorer progression-free survival (PFS) in the all-targeted therapy group and the group treated with sorafenib alone [20]. The first-line Bevacizumad and platinum based chemotherapy for patients with advanced lung adenocarcinoma, the patients with KRAS^G12D^ mutation had significantly poorPFS and OS than patients with wild-type KRAS or other KRAS mutations [21]. Studies reported that these defective KRAS mutation proteins showed different affinities for downstream effectors and resulted in unique biological and clinical behaviors [20,22,23]. However, there is still an unmet need for exploring the detailed regulatory mechanisms that can explain the different phenotypes of metastasis induced by KRAS^G12V^ and KRAS^G12D^.

The Ras homologous (Rho) GTPase family is a branch of the Ras superfamily and contains more than 20 members. The best-characterized proteins of Rho family are RhoA, Cdc42 and Rac1. The Rho family proteins regulate various cellular processes, including cytoskeleton rearrangement, cell polarity, cell contractility, cell cycle and gene expression [24,25,26,27]. It has become increasingly evident that Rho family proteins also play important roles in cancer progression, inflammation, wound repair and metastasis [28,29]. Furthermore, the effects of the crosstalk between Ras and Rho GTPases on cell transformation and cell migration were also reported in several studies [30,31,32,33].

In this study, we hypothesized that the mechanisms of NSCLC metastasis driven by KRAS^G12V^ and KRAS^G12D^ are distinct. First, we observed that the mutations of KRAS^G12V^ and KRAS^G12D^ induced distinct cell morphology changes, contrasting cell functions and divergent epithelial–mesenchymal transition (EMT) markers in H838 isogenic cells. In vivo metastasis model, the mutation of KRASG12V and KRASG12D were showed different abilities to metastasize and the more aggressive metastatic pattern was observed in the group of KRASG12V mutation. Then we set out to identify the regulatory pathway that involved in the metastasis of H838KRAS^G12V^ cells and H838KRAS^G12D^ cells. Our Gene Set Enrichment Analysis (GSEA) results of H838KRAS^G12V^ cells presented that KRAS^G12V^ was negatively correlated with RhoA-related pathways and mitogen-activated protein kinase (MAPK) pathway. The lower activation of RhoA and higher activation of Wnt/β-catenin was detected in H838KRAS^G12V^ cells. In contrast, the higher activation of RhoA and lower activation of Wnt/β-catenin was probed in H838KRAS^G12D^ cells. Next, we reversed the expression of RhoA and detected the restored activation of Wnt/β-catenin in H838KRAS^G12D^ cells. In addition, the migration ability of H838KRAS^G12V^ cells was also retrained by Wnt inhibitor LGK974. We also further evaluated the inhibitory effect of Wnt inhibitor LGK974 on KRAS^G12V^ (RERF-LC-AD2, H441, H2444) and KRAS^G12D^ cancer cells (SK-LU-1). The migration ability of RERF-LC-AD2 and H441 were restrained, but migration ability of SK-LU-1 was increased after treatment with LGK974.

Our studies demonstrate that RhoA acts as an upstream regulator of Wnt/β-catenin signaling and the axis is an important regulatory mechanism in NSCLC metastasis induced by KRAS^G12V^ and KRAS^G12D^ mutations. These findings may be particularly important in targeting NSCLC metastasis induced by KRAS^G12V^ and KRAS^G12D^ mutations in the future.

## 2. Results

### 2.1. Different KRAS Mutation Subtypes Cause Distinct Cell Morphologies and Characteristics

**Figure 1:**
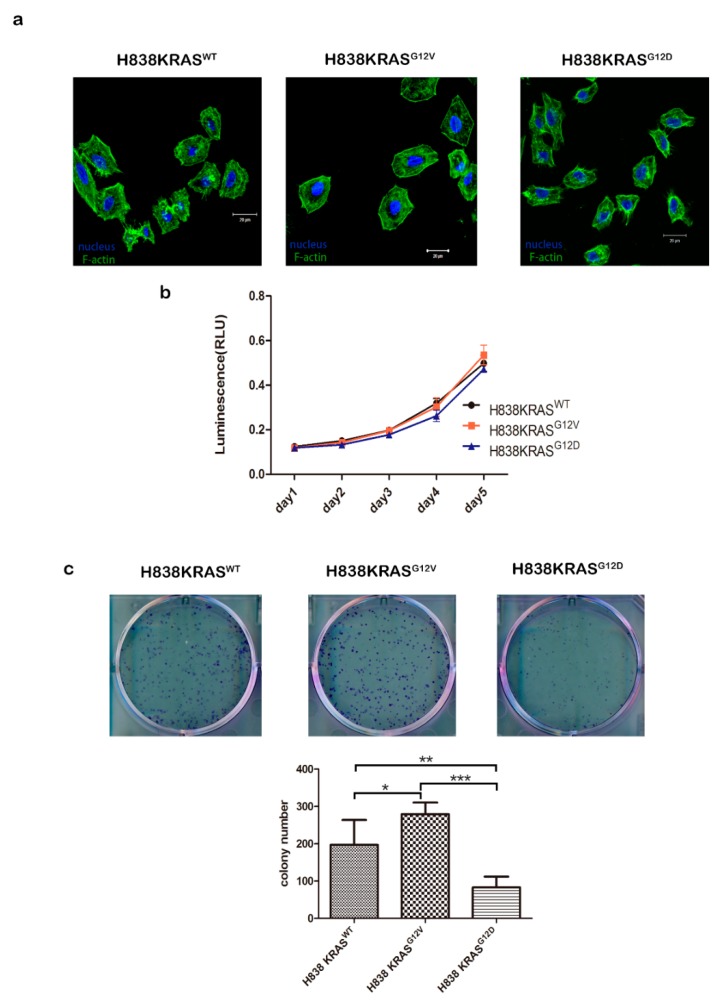
KRAS^G12V^ and KRAS^G12D^ mutations do not affect cell proliferation. (**a**) Immunofluorescence (IF) staining of F-actin in H838 KRAS isogenic cell lines. F-actin is visualized by fluorescent green phalloidin-staining Acti-stain™ 488. DAPI is the blue nuclear stain. Immunoreactivity was captured by confocal microscopy. (**b**) The cell proliferation of the H838 KRAS isogenic cell lines are the same. Cell proliferation was measured using a CellTiter-Glo Luminescent Cell Viability assay. (**c**) The ability of colony formation in H838 KRAS isogenic cell lines. The cell colonies were stained with crystal violet and counted 7 days after cell seeding. The values represent the means ± s.d. of three independent assays (*n* = 3, * *p* < 0.05, ** *p* < 0.01, *** *p* < 0.001).

**Figure 2:**
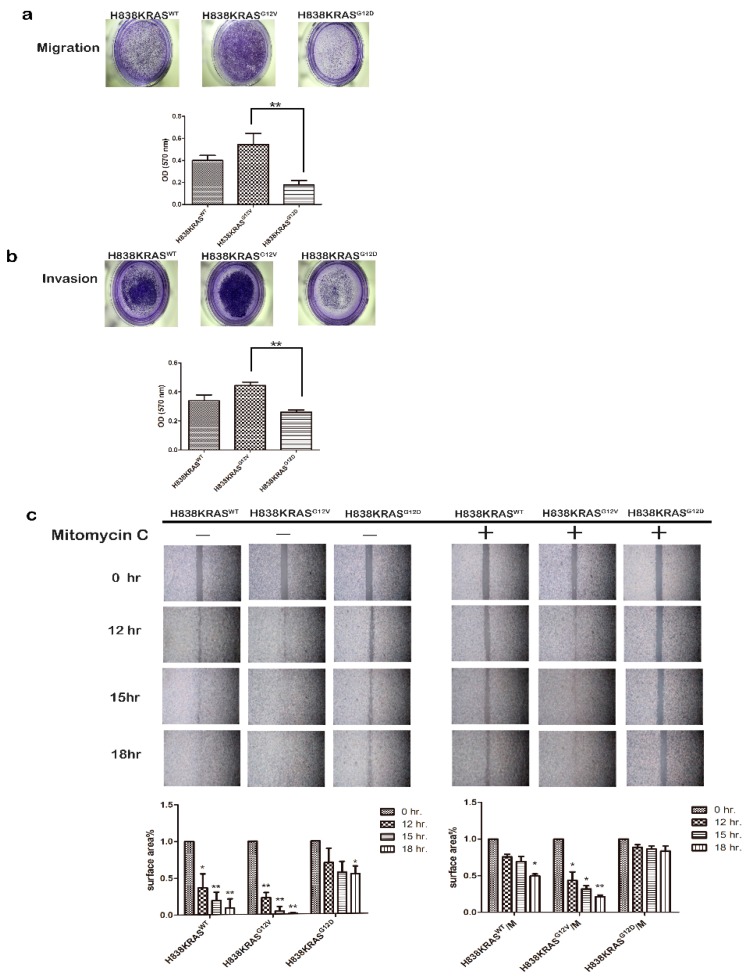
KRAS^G12V^ confers greater oncogenic ability than KRAS^G12D^. (**a**) The ability of migration in H838 KRAS isogenic cell lines. (**b**) The ability of invasion in H838 KRAS isogenic cell lines. The ability of migration and invasion were assessed by a Transwell system. The cells were stained with crystal violet. The OD ratio of crystal violet was measured. The values represent the means ± s.d. of three independent assays (*n* = 3, * *p* < 0.05 and ** *p* < 0.01). (**c**) Representative image and quantification of wound healing assays in the H838 KRAS isogenic cell lines. Mitomycin C (10 μg/mL) was used to inhibit cell proliferation. The values represent the means ± s.d. of three independent assays (*n* = 3, * *p* < 0.05 and ** *p* < 0.01).

To minimize effects caused by genetic background heterogeneity, we used a series of three isogenic lung cancer cell lines, namely, H838KRAS^WT^, H838KRAS^G12V^ and H838KRAS^G12D^ (Figure S1a–c). First, we observed obvious morphology changes in H838KRAS^G12D^ cells by immunofluorescence (IF) staining of F-actin. The smaller cell size and more microspikes were observed in H838KRAS^G12D^ cells than H838KRAS^WT^ and H838KRAS^G12V^ cells (Figure 1a). However, the KRAS^G12V^ and KRAS^G12D^ did not affect cell proliferation (Figure 1b). To differentiate the distinct impact of KRAS^G12V^ and KRAS^G12D^ mutations on cell functional effects, several cell functional assays were performed. The H838KRAS^G12V^ cells showed a greater ability of colony formation than H838 KRAS^G12D^ cells (Figure 1c, * *p* < 0.05 and ** *p* < 0.01). The mutation of KRAS^G12V^ conferred greater abilities of migration (Figure 2a, ** *p* < 0.01) and invasion (Figure 2b, ** *p* < 0.01) than KRAS^G12D^ mutation. To clarify the effect of migration and proliferation, cells were treated with a proliferation blocker Mitomycin C (10 μg/mL). The cells with KRAS^G12V^ mutation improved the ability of wound healing even after treatment with Mitomycin C (Figure 2c). Together, these findings suggest that KRAS^G12V^ and KRAS^G12D^ mutations might affect cell functions related to metastasis but not cell proliferation.

### 2.2. The Mutation of KRAS^G12V^ Confers a Greater Capacity in Metastasis

**Figure 3:**
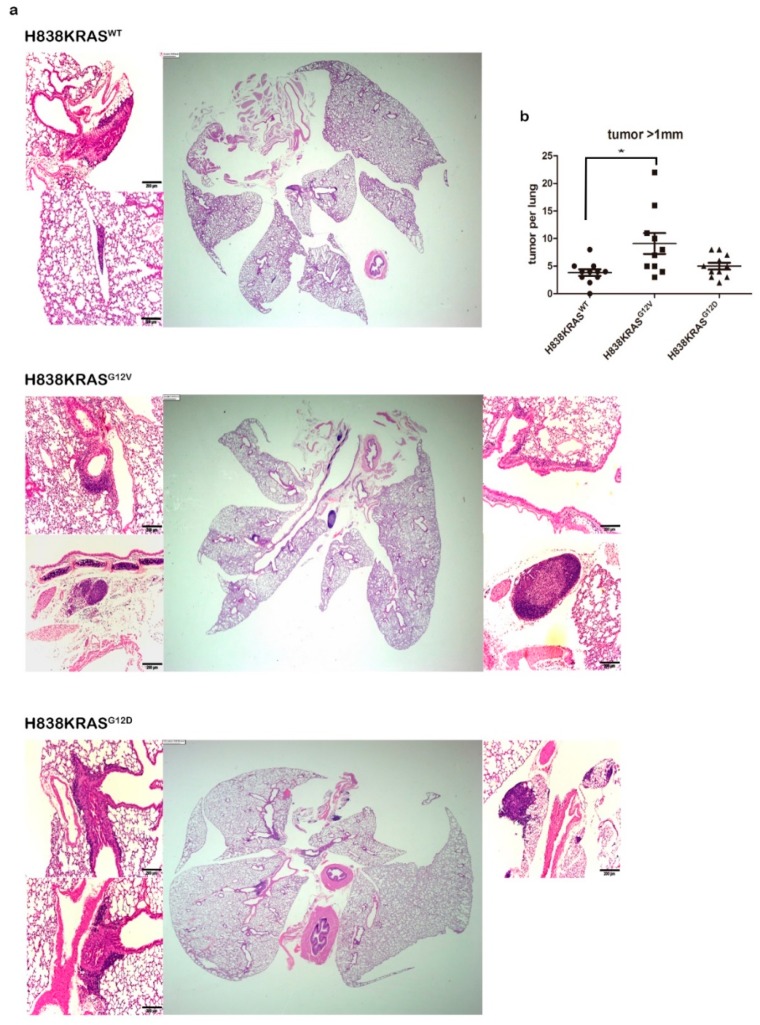
KRAS^G12V^ mutation induced a greater amount of metastatic nodules in mice lung tissues. (**a**) Lung sections of mice stained with H&E. (**b**) The 10 lung sections from each mouse were examined. The average number of metastatic nodules in the 10 sections was acquired. The histograph showing the Mann–Whitney U statistical result of the group of KRAS^WT^ (*n* = 11), the group of KRAS^G12V^ (*n* = 10) and the group of KRAS^G12D^ (*n* = 11), Scale bar 200 µm, * *p* < 0.05.

Next, we established a murine metastasis model to assess the metastatic potential of KRAS mutations in Vivo. The H838 isogenic cells were injected into the nude mice through tail vain. The lung tissues of mice were removed after 140 days injection and immediately preserved in 10% formalin. We further performed a histopathological examination of the formalin-fixed lung tissues to confirm the presence of micrometastases. The KRAS^G12V^ mutation induced a greater amount of micrometastases in mice lung tissues (Figure 3a) and the results showed statistical significances compared to KRAS^WT^ (Figure 3b, * *p* < 0.05). Thus, these data show that KRAS mutations are capable of different metastatic potential and KRAS^G12V^ mutation confers a more aggressive phenotype.

### 2.3. The Distinct Different Expression Profiling in KRAS^G12V^ and KRAS^G12D^ Mutation

**Figure 4:**
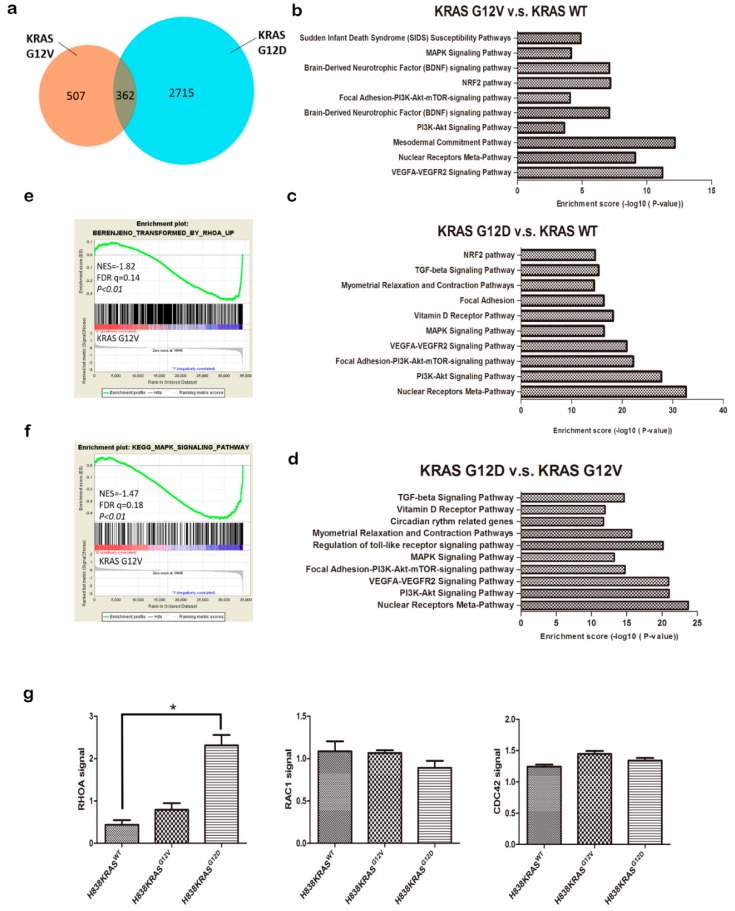
Microarray analysis of differentially regulated genes between H838 KRAS^G12V^ and KRAS^G12D^ cells. (**a**) Venn diagram representing the number of genes that was differentially expressed in H838 KRAS^G12V^ and KRAS^G12D^ cells with a fold change of 2.0 and a false discovery rate of 0.05. (**b**) Number and categories of genes up- and downregulated in H838 KRAS^G12V^ cells. (**c**) Number and categories of genes up- and downregulated in H838 KRAS^G12D^ cells. (**d**) Number and categories of genes up- and downregulated in H838 KRAS^G12V^ vs. H838 KRAS^G12D^ cells. The number of genes expressing a twofold or greater significant difference (*p ≤* 0.05) is plotted for the listed categories. (**e**) The correlation between RhoA pathway and KRAS^G12V^ by Gene Set Enrichment Analysis (GSEA). (**f**) The correlation between MAPK pathway and KRAS^G12V^ by GSEA. The normalized enrichment score (NES) and *p* value are shown. (**g**) The activation of Rho GTPase family proteins (RhoA, Cdc42, and Rac1) in H838 KRAS isogenic cell lines was evaluated by G-LISA assays. The values represent the means ± s.d. of five independent assays (*n* = 5, * *p* < 0.05 and ** *p* < 0.01).

To determine the molecular mechanisms that might be regulated by KRAS mutations, we investigated transcriptome changes and related signaling pathway by using an Affymetrix microarray. The microarray data were collected and analyzed for the significant differential expression of genes by Affymetrix Transcriptome Analysis Console (TAC) (*p* ≤ 0.05 and ± ≥ 2 fold change). We observed the number of significantly differentially expressed genes is 2715 in H838 KRAS^G12D^ cells and 507 in H838 KRAS^G12V^ cells (Figure 4a). To gain insights into the modulated genes, we clustered them in pathways and sorted them according to their biological function. We highlighted the top 10 ranked pathways of the most significant difference (Figure 4b,c) and gene counts (Figure S2a,b) that were involved in by KRAS^G12V^ or KRAS^G12D^ modulated gene sets. We also identified the top 10 ranked pathways of the most significant difference and the gene counts between in H838 KRAS^G12V^ and H838 KRAS^G12D^ cells (Figure 4d and Figure S2c). These results reveal that KRAS^G12V^ and KRAS^G12D^ govern distinct gene profiling and signaling pathways.

### 2.4. The KRAS^G12D^ Induce RhoA Activation But Not KRAS^G12V^

To explore the distinct regulatory networks of KRAS mutations, we executed the Gene Set Enrichment Analysis (GSEA). The GSEA identified the clear negative correlation between the sets of genes that are transformed by RhoA and the KRAS^G12V^ mutation (FDR = 0.14, *p* < 0.01, Figure 4e). RhoA, CDC42 and RAC1 are members of Rho GTPase family, they are key regulators in rearrangement of cell cytoskeleton and cell contractility. We further evaluated the activity of Rho family GTP-binding proteins. Both activities of CDC42 and RAC1were showed no differences in the H838 KRAS isogenic cell lines by G-LISA (Figure 4g, middle and right). However, we detected a higher level of RhoA activation in H838 KRAS^G12D^ cells than in H838 KRAS^G12V^ cells (Figure 4g, left, * *p* < 0.05).

The GSEA also identified that the gene expression signatures of H838KRAS^G12V^cells are negetively associated with the gene set of “KEGG MAPK SIGNALING PATHWAY” (FDR = 0.18, *p* < 0.01, Figure 4f). Next, we probed the protein level of MAPK related molecules, the phosphorylation of extracellular signal-regulated kinases (ERK) and c-Jun N-terminal kinase (JNK) and the results were showed the obvious differences in H838 KRAS^G12D^ cells (Figure S3). Taken together, KRAS^G12V^ showed a negative correlation of RhoA and MAPK pathway. These results further suggest that KRAS^G12V^ and KRAS^G12D^ participated in different cytoskeletal regulatory functions.

In addition, both of microarray and western blotting exhibited the upregulated expression of guanine nucleotide exchange factor H1 (GEF-H1) in H838 KRAS^G12D^ cells (Figure S4a,b). GEF-H1, a guanine nucleotide exchange factor for RhoA [34], is up regulated and critical for supporting cell survival and growth in RAS-induced transformation of Mouse Embryonic Fibroblasts (MEF) cells, xenograft model and tissue sections of pancreatic adenocarcinoma [35,36]. GEF-H1 was also identified as an upstream regulator of RhoA and ERK effector that govern cell protrusion dynamics [37,38,39]. Furthermore, the activated pattern of MAPK signaling were also probed in H838 *KRAS^G12D^* cells, and the association between RhoA and MAPK signaling was reported by several studies [40,41,42]. These data support the correlation that KRAS^G12D^ may arouse activation of RhoA through upregulation of GEF-H1, but KRAS^G12V^ could not.

### 2.5. The H838 KRAS^G12D^ Cells Displayed Downregulated Levels of β-Catenin

**Figure 5:**
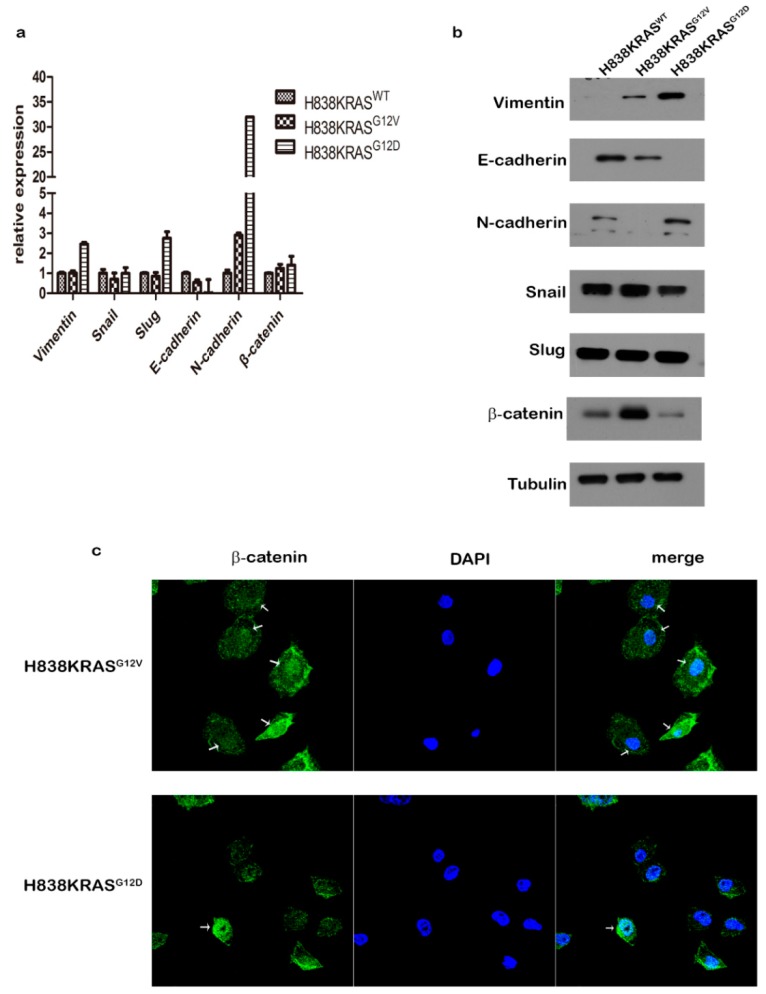
The expression profiles of epithelial-mesenchymal transition (EMT)-related genes in H838 KRAS isogenic cell lines. (**a**) The relative RNA expression levels of EMT-related genes were evaluated by real-time PCR. (**b**) Immunoblot analysis of EMT-related genes. Tubulin was used as an internal control. (**c**) The IF staining of β-Catenin in H838 KRAS isogenic cells. The β-Catenin is visualized with fluorescence staining. DAPI is nuclear stain. Immunoreactivity was demonstrated by confocal microscopy. The results of realtime PCR represent the means ± s.d. of three independent assays (*n* = 3). The uncropped blots and molecular weight markers are shown in Figure S9.

Cancer metastasis is the highest cause of cancer related death. The epithelial-mesenchymal transition (EMT) process is a well described mechanism of metastasis that confers migratory and invasive properties or stem cell-like features that allow cells to disseminate from primary sites to secondary sites by releasing into blood or lymph vessels. Due to the differential capacity of migration in our results, we evaluated the expression profiling of EMT markers in these isogenic cells. We found that the KRAS^G12D^ mutation downregulated the expression of E-cadherin and upregulated the expression of N-cadherin at the RNA level (Figure 5a). In protein level of EMT markers, KRAS^G12V^ mutation increased the expression of vimentin and β-catenin but decreased the expression of E-cadherin and N-cadherin. In contrast, KRAS^G12D^ mutation promoted the expression of vimentin and N-cadherin but abolished the expression of E-cadherin (Figure 5b).

**Figure 6:**
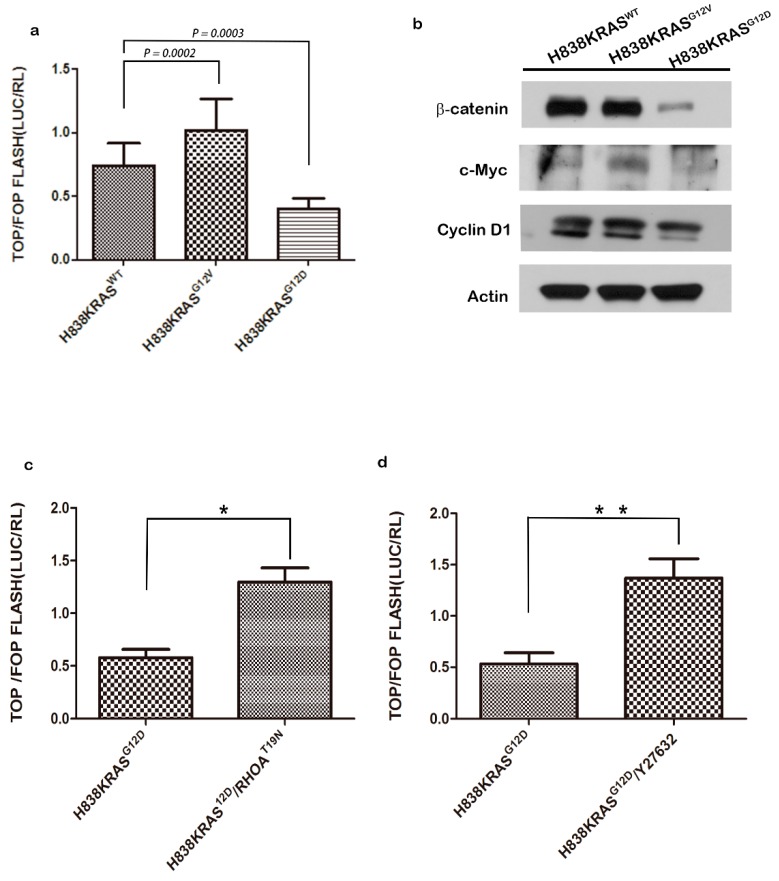
The inactivation of RhoA restores the activity of Wnt/β-catenin pathway. (**a**) The activity of Wnt/β-catenin in H838KRAS isogenic cells as measured by TCF/LEF reporter assays. (**b**) The western blotting of Wnt/β-catenin pathway-related genes. β-actin was used as an internal control. (**c**) The activity of Wnt/β-catenin after inactivation of RhoA by transfecting with RhoA^T19N^ plasmids in H838KRAS^G12D^ cells. (**d**) The activity of Wnt/β-catenin after inactivation of RhoA by treating with Y27632 inhibitor in H838KRAS^G12D^ cells. The activity of Wnt/β-catenin were access by TCF/LEF reporter assays. All reporter activities were evaluated by a dual luciferase system, and the values represent the means ± s.d. of six independent assays (*n* = 6, * *p* < 0.05 and ** *p* < 0.01). The uncropped blots and molecular weight markers are shown in Figure S10.

Notably, the decreased protein level of β-catenin was observed in H838KRAS^G12D^ cells but not in the RNA level. To confirm this finding, we probed the translocation of β-catenin by IF staining in H838 KRAS isogenic cells. The result of IF staining showed that H838KRAS^G12D^ cells exhibited a lower distribution of β-catenin in the nucleus than did H838KRAS^G12V^ cells (Figure 5c). The T cell factor/lymphoid enhancer factor (TCF/LEF) reporter assays were also performed to define the activation status of β-catenin. We found that the quenched activation of β-catenin in H838 KRAS^G12D^ cells compared to H838KRAS^WT^ and H838KRAS^G12V^ cells (Figure 6a). Moreover, we detected the expression of several downstream effectors in Wnt related signaling pathways by immunoblotting. The immunoblotting results showed a lower expression of total β-catenin, c-myc and cyclin D1 in H838 KRAS^G12D^ cells (Figure 6b).

To validate the correlation between KRAS mutation and β-catenin, we transfected exogenous DsRed-tagged wild-type (WT) KRAS, KRAS G12V and KRAS G12D into MRC-5 normal human lung fibroblast cells. In the image of IF staining, the white arrows indicate the transfected cells, and the yellow arrows indicate the nontransfected cells. The staining results emphasize that KRAS G12D-transfected cells displayed a lower distribution of β-catenin in the nucleus, but a greater amount of β-catenin accumulated in the nucleus of KRAS G12V-transfected cells (Figure S5). Taken together, these data indicate that KRAS^G12D^ attenuated the localization of β-catenin in nucleus.

### 2.6. Activation of RhoA Suppresses the Activation of Wnt in H838 KRAS^G12D^ Cells

As has been previously reported, inactivation of RhoA may promote the translocation of β-catenin into nucleus and increase the size and number of adenomas in colon cancer [43,44]. To dissect the regulatory correlation of RhoA and Wnt/β-catenin, we performed immunoblotting and observed a slightly increased total of β-catenin after treatment with Y23762 (RhoA inhibitor) in H838 KRAS^G12D^ cells (Figure S6). To define the direct correlation between RhoA and activation of β-catenin, we transduced dominant-negative form of RhoA (RhoA^T19N^) into these cells or treated cells with Y23762 treatment. The results showed that H838KRAS^G12D^ cells exhibited an approximately threefold restoration of Wnt/β-catenin activation after introducing RhoA^T19N^ (Figure 6c, * *p* < 0.05). We also observed a nearly threefold restoration of β-catenin activation after treated H838KRAS^G12D^ cell with Y23762 (Figure 6d, ** *p* < 0.01). Collectively, our findings prove the correlation of KRAS/RhoA/β-catenin and RhoA act as a directly upstream regulator of Wnt/β-catenin signaling.

### 2.7. The Mutation of KRAS^G12V^ Promote Metastasis Through Wnt Pathway

**Figure 7:**
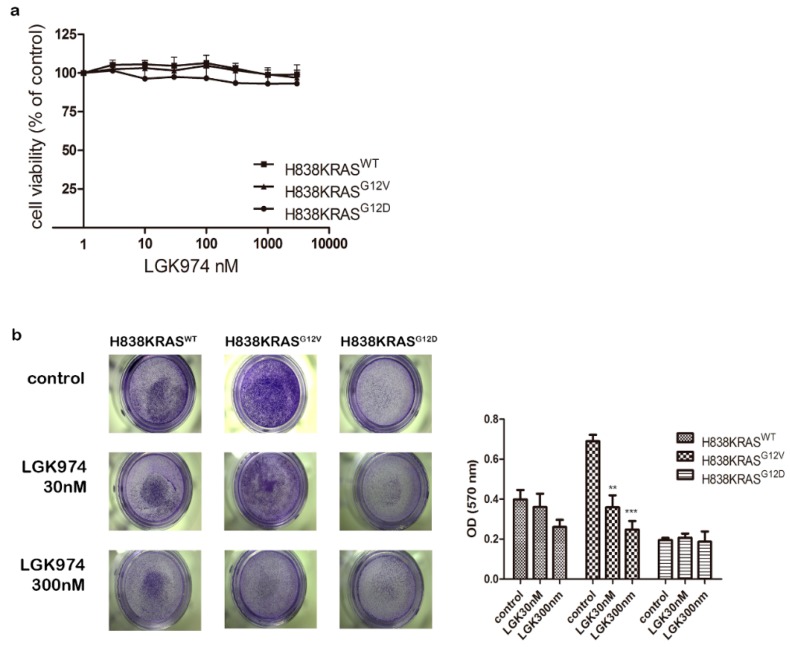
The Wnt inhibitor LGK974 suppress the migration ability of H838KRAS^G12V^ cells. (**a**) The cell viability assays of H838KRAS isogenic cells after treating with LGK974. (**b**) The migration assays of H838KRAS isogenic cells after treating with no LGK974 (control), 30 nM LGK974 and 300 nM LGK974. The OD ratio of crystal violet was measured. The values represent the means ± s.d. of five independent assays (*n* = 5, ** *p* < 0.01 and *** *p* < 0.001).

To determine the effect of Wnt pathway on cell proliferation, we performed cell proliferation assays under Wnt inhibitor (LGK974) treatment and found that LGK974 had no effect on cell viability (Figure 7a). Second, to evaluate inhibitory effects of cell migration under LGK974 treatment, we treated H838KRAS^G12V^ cells with 30 nM and 300 nM of LGK974 and then performed migration assays. The results of migration assay showed that both 30 nM and 300 nM of LGK974 restricted migration ability of H838KRAS^G12V^ cells and the inhibitory effect achieved statistical significance (Figure 7b, * *p* < 0.05 and ** *p* < 0.01). To further prove the inhibitory effect of LGK974 on other KRAS-mutant NSCLC cells, we tested several KRAS^G12V^ mutant NSCLC cells (RERF-LC-AD2, H2444, H441) and KRAS^G12D^ mutation cells (SK-LU-1) (Figure S7a,b). The result indicated that the treatment of 20 nM LGK974 inhibited migration ability of RERF-LC-AD2 and H441 (* *p* < 0.05) and slightly inhibit migration ability of H2444 (Figure S8a–c). In contrast, the treatment of 20nM LGK974 promoted migration ability of SK-LU-1 (* *p* < 0.05) (Figure S8d). Together, these data support the idea that Wnt/β-catenin pathway has potent effects on migration in KRAS^G12V^-mediated metastasis.

## 3. Discussion

In this study, we sought to elucidate the different mechanisms of RhoA/Wnt in NSCLC metastasis elicited by KRAS^G12V^ and KRAS^G12D^ mutations. First, we found the KRAS^G12V^ mutation induced a more aggressive metastasis phenotype both in vitro and in vivo. Next, we identified the activation of WNT/β-catenin by KRAS^G12V^ mutation resulted in a more aggressive metastasis phenotype. The weaker metastasis phenotype of KRAS^G12D^ mutation may result from the activation of RhoA and the RhoA further suppress WNT/β-catenin signaling. The treatment of Wnt inhibitor LGK974 showed inhibitory effect on the migration of H838KRAS^G12V^ cells, RERF-LC-AD2 and H441 cells. Our findings explain the distinct oncogenic effects of RhoA/WNT pathway conferred by KRAS^G12V^ and KRAS^G12D^ mutations.

Malignant transformation by RAS requires the concomitant activation of downstream effectors, including Rho GTPase family [31,45]. RhoA may play contradictory roles in tumorigenesis; several studies have attributed tumor suppressor characteristics to RhoA. Two of the studies demonstrated that RhoA was negatively associated with AKT phosphorylation and cyclin D1 in both endothelial cells and KRAS-driven adrenocortical cancer cell lines [46,47]. Moreover, when inactivation of RhoA signaling by transducing dominant-negative form of RhoA resulted in larger adenomas and decreased survival in a zebrafish model of *KRAS*-induced hepatic adenoma by rising AKT/S6 signaling and cyclin D1 [48]. In addition, the reduction of RhoA was associated with lymph node metastasis and shorter survival in an analysis of 137 colorectal tumor samples [49]. Our findings are in line with this research, and we suggest that RhoA may acts as a tumor suppressor in KRAS induced NSCLC.

However, the correlation between the family of RHO GTPase and β-catenin in NSCLC metastasis remains unclear. The β-catenin acts not only as a transcriptional factor but as a regulator of cell–cell junctions; β-catenin sustains cell–cell junctions by binding to α/β-catenin and to the cytoplasmic domain of E-cadherin [50,51,52,53]. The KRAS^G12V^ accelerate tumor growth and reinforce stem-like properties of cancer cells through activates canonical Wnt signaling [54]. In this work, we identify β-catenin as a downstream effector of RhoA in KRAS mutations mediated NSCLC metastasis. When inactivation of RhoA by RhoA inhibitor or by transfecting with RhoA^T19N^ plasmid, we observed the restoration of β-catenin activation in H838KRAS^G12D^ cells. Rodrigues, P. et al. demonstrated that RhoA was as a novel tumor suppressor in colorectal cancer. When RhoA is inactivated, the nuclear translocation of β-catenin is increased and then Wnt/β-catenin signaling could be aroused in human colon cancer cells. Moreover, the inactivation of RhoA resulted in larger and more numerous adenomas and decreased survival in an APC mutation-driven murine colon cancer model [43]. In addition, the coactivation of Wnt/β-catenin signaling and KRAS^G12D^ mutation could accelerate the development of a more aggressive phenotype in a KRAS^G12D^ mutation mouse model [44]. These findings are in agreement with ours, and our findings prove the correlation between *KRAS*/RhoA/Wnt/β-catenin signaling in metastasis of NSCLC. Nevertheless, the lack of clinical evidence to strengthen the correlation of KRAS/RhoA/Wnt is the limitation of our study. In the further studies, the establishment of the Kras-driven genetically engineered mouse models and the collection of clinical samples of KRAS-mutant lung adenocarcinoma may provide more evidence.

In summary, our data provide the important insight that the regulatory mechanisms of KRAS^G12V^/KRAS^G12D^-mediated metastasis are different. We are the first to demonstrate RhoA act as a key role in modulating the activation of Wnt/β-catenin pathway in NSCLC metastasis. We elucidate the distinct regulatory of RhoA/Wnt/β-catenin pathways in NSCLC metastasis induced by KRAS^G12V^ and KRAS^G12D^. To improve treatment efficacy of KRAS^G12V^ mutation mediated metastasis, we suggest that the activation of Wnt/β-catenin signaling pathway should be inhibited. The detailed regulatory molecules involved in the interaction of KRAS/RhoA/Wnt/β-catenin signaling still need to be explored more clearly. It will be also important that future research investigate other specific aspects of KRAS mutations-induced cancers. Lipford JR et al. reported that the treatment with AMG510 (the inhibitor of KRAS G12C) or combined treatment with AMG510 and anti-PD-1 may provoke immunity against tumor antigen [55]. Future studies could investigate the association between the immune response and immune microenvironment induced by inhibition of KRAS.

## 4. Materials and Methods

### 4.1. Cell Culture

Isogenic cell pairs H838KRAS^WT^, H838KRAS^G12V^ and H838KRAS^G12D^ were purchased from Horizon Discovery (Cambridge, UK). RERF-LC-AD2, H441, SKLU-1 and H2444 were purchased from ATCC. All cell lines were grown in Roswell Park Memorial Institute (RPMI) supplemented with 10% Fetal Bovine Sera (FBS), 100 U/mL penicillin and 100 mg/mL streptomycin (Thermo Fischer Scientific, Waltham, MA, USA) except for MRC-5, which was cultured in MEM supplemented with 10% FBS, glutamine, penicillin and streptomycin (Thermo Fischer Scientific, Waltham, MA, USA). Cells were maintained at 37 °C in a humidified atmosphere containing 5% CO_2_. All cell lines were used at early passages (<3 months after resuscitation of the original cells, between passages 7 and 30) for these experiments. The KRAS status for all cell lines used was obtained from the Catalogue of Somatic Mutations in Cancer (COSMIC) database, version 84 (http://cancer.sanger.ac.uk/cosmic).

### 4.2. Plasmids

Plasmids encoding the *RhoA^T19N^* mutant (plasmid no. 12967) were purchased from Addgene. KRAS WT, KRAS G12V and KRAS G12D were subcloned into pDsRed1-N1 (CLONTECH, catalog no. 921-1).

### 4.3. RT-qPCR

Total RNA was extracted from cells using a NucleoSpin^®^ RNA kit (Macherey-Nagel, Düren, Germany) and was quantified by a NanoDrop (Thermo Fischer Scientific, Waltham, MA, USA). Reverse transcription was performed using a GoScript™ Reverse Transcription System (Promega, WI, USA). For qPCR analysis, TaqMan Real-Time PCR Master Mixes were used (Thermo Fischer Scientific, Waltham, MA, USA), and amplification was performed on a 7500 Real-Time PCR System. To normalize for cDNA loading, GAPDH was used as an internal control. The PCR primers used in this study are listed in Table S1.

### 4.4. Microarray and Data Mining

RNA samples were isolated from H838KRAS isogenic cell lines for microarray analysis using the NucleoSpin^®^ RNA kit (Macherey-Nagel, Düren, Germany). RNA samples from three biological replicates were used for the microarray experiment, which was conducted by the Affymetrix Gene Expression Service Lab at Academia Sinica, Taipei, Taiwan (http://ipmb.sinica.edu.tw/affy/). Gene expression profiles were determined using Affymetrix Clariom D GeneChips (Affymetrix Inc., Santa Clara, CA, USA) according to the manufacturer’s instructions. GSEA was performed by the JAVA program (http://www.broadinstitute.org/gsea) using MSigDB gene set collections and was visualized in Enrichment Map software. Microarray data were deposited in the Gene Expression Omnibus (GEO) database (GSE119146).

### 4.5. Immunofluorescence Staining

Cells were grown on 12-mm round coverslips in 24-well plates. The coverslips were rinsed 3 times in PBS, fixed in 4% formaldehyde (Sigma-Aldrich, St. Louis, MO, USA) at room temperature for 10 min, and permeabilized with 0.5% Triton X-100 in PBS for 5 min. After blocking in 5% BSA/PBS for 1 h, the coverslips were incubated with an anti-β-catenin antibody (Cell Signaling Technology, Danvers, MA, USA 1:100) overnight followed by incubation with a fluorescence-conjugated secondary antibody (GeneTex, Irvine, CA, USA) at a concentration of 1:1000 for 1 h at room temperature. Following additional washes with PBS, cells were stained with 4’,6-diamidino-2-phenylindole (DAPI; Invitrogen Molecular Probes, cat. no. D1306). For F-actin visualization, cells were stained with Acti-stain 488 fluorescent phalloidin (Cytoskeleton, Inc., Denver, CO, USA). After aspirating and washing the cells with PBS, the coverslips were inverted and mounted to glass slides using Fluoro-Gel (Electron Microscopy Sciences, Hatfield, PA, USA, catalog no. 17985-10). Images were obtained with a Zeiss UV LSM510 META laser-scanning confocal microscope (Carl Zeiss, Jena, Germany).

### 4.6. Immunoblotting and Reagents

Cells were lysed in RIPA buffer (Cell Signaling Technology, Danvers, MA, USA) and supplemented with protease and phosphatase inhibitors (Thermo Fischer Scientific, Waltham, MA, USA). The extracted proteins were quantified using a Pierce BCA Protein Assay kit (Thermo Fischer Scientific, Waltham, MA, USA). Twenty micrograms of total protein were then subjected to SDS-PAGE on 10%–12%% gels and was then transferred to Hybond ECL nitrocellulose membranes (GE Healthcare, Milwaukee, WI, USA). Membranes were blocked in TBST (Tris-buffered saline, 10 mM Tris-HCl, pH 7.4; 150 mM NaCl; and 0.1% Tween 20) plus 5% nonfat dry milk for 1 h, and the membrane was then probed with the indicated primary and secondary antibodies. Following washes with TBST, membranes were developed using Immobilon Western Chemiluminescent Horseradish peroxidase (HRP) Substrate (Millipore, Billerica, MA, USA). The antibodies used in this study are listed in Table S2. Other chemicals were obtained from the following suppliers: Sigma (puromycin), Cell Signaling Technology (EGF) and Selleckchem (Houston, TX, USA) (Y-27632 2HCl and LGK-974), and Thermo Fisher Scientific (Lipofectamine 3000).

### 4.7. Cell Viability Assay

A CellTiter-Glo Luminescent Cell Viability Assay kit (Promega, Madison, WI, USA) was used to determine cell proliferation. Cells were seeded in 96-well plates (1000 cells/well) and incubated for the indicated time. Equal volumes of the CellTiter-Glo reagents were added into each well and follow up with 10 min room temperature incubation. The luminescence signals were recorded using a microplate luminescence reader (Victor X4 Multilabel Plate Reader, PerkinElmer Life and Analytical Science, Turku, Finland).

### 4.8. Migration, Invasion Assay and Colony Formation

For the migration assay, 5 × 10^4^ cells were counted and plated into the top chamber of Transwell inserts (Corning, Nemaha, KS, USA) in serum-free medium, and the medium in the bottom chambers was supplemented with 10% FBS. Invasion assays were accomplished by Transwell inserts coated with growth factor reduced (GFR)-Matrigel (BD Biosciences, #356230). For the invasion assay, 1 × 10^5^ cells were counted and plated into the top chamber of Transwell inserts (Corning). After overnight incubation, cells were fixed and stained with 0.1% crystal violet solution. The migrated and invaded cells were observed by a microscope. The fixed cells in the underside were dissolved in 200 μL of 33% acetic acid; the absorbance was measured at 570 nm by microplate reader. Each sample was analyzed in triplicate, and three independent experiments were performed. For the colony formation assay, 500 cells were counted and plated into the 6-well plate for 7 days. The colonies were stained with crystal violet and counted.

### 4.9. Wound Healing Assay

H838KRAS isogenic cell lines were seeded into the designated areas of ibidi Culture-Insert 2 Well dishes (ibidi, GmbH, Munich, Germany). After cell attachment, the culture insert was gently removed, and a 500-μm-wide cell-free gap (wound) was generated. Cells were treated with 10 μg/mL Mitomycin C for 2 h prior to wounding. Images were captured at the indicated times. Cell motility was quantified by measuring the wound coverage at each time point. The area of every wound at each time point was then normalized to its respective area at time 0. All experiments were carried out in triplicate and repeated three times.

### 4.10. GTPase Activation Assay

The activity of RhoA/Rac1/Cdc42 was determined using a RhoA/Rac1/Cdc42 G-LISA Activation Assay kit (BK135) as recommended by the manufacturer (Cytoskeleton, Inc., Denver, CO, USA). Briefly, the samples were added to the RhoA/Rac1/Cdc42 G-LISA plate, and the plate was placed on a microplate shaker operating at 400 rpm and was incubated at 4 °C for 30 min. After washing three times, anti-RhoA/anti-Rac1/anti-Cdc42 primary antibodies were added to each well, and the plate was placed on the shaker for 45 min. After washing three times, 50 µL of diluted HRP-labeled secondary antibodies were added to the wells, and the plate was placed on the shaker at room temperature for 45 min. The wells were washed, 50 µL of HRP detection reagent was added to the wells, and the luminescence signal was detected using a microplate luminescence reader (Victor X4 Multilabel Plate Reader, PerkinElmer Life and Analytical Science, Turku, Finland). The results are shown as the relative luminescence units (RLUs) with respect to the background luminescence.

### 4.11. Luciferase Reporter Assay

Cells were transfected with TOPFlash (Addgene #12456), FOPFlash (Addgene #12457) or pGL4.10 (Promega) luciferase reporter plasmids with Lipofectamine 3000 (Thermo Fischer Scientific, Waltham, MA, USA). Firefly and Renilla luciferase activities were determined 24 h after transfection using the Dual-Glo Luciferase Assay System (Promega). The firefly reporter is measured first by adding Luciferase Assay Reagent II to generate a luminescent signal. After evaluating the firefly luminescence, the Renilla luciferase reaction is simultaneously initiated by adding Stop & Glo Reagent. The relative reporter activity was obtained by normalization to Renilla luciferase activity (ratio of firefly luciferase activity to Renilla luciferase activity). Each assay condition was performed in triplicate.

### 4.12. Animal Experiments

All experiments involving animals followed the regulatory standards approved by the National Taiwan University College of Medicine and College of Public Health Institutional Animal Care and Use Committee (IACUC) (IACUC approval number: 20160374). For the experimental metastasis model, cells were resuspended (5 × 10^5^ cells in 100 μL of PBS) and intravenously injected through the tail vein of nude mice, and observed for 140 days (National Laboratory Animal Center, NLAC). The group of KRAS^WT^ (*n* = 11), the group of KRAS^G12V^ (*n* = 10, one died before sacrificing), and the group of KRAS^G12D^ (*n* = 11). Paraffin-embedded sections of lungs from the mice of all groups were stained with H&E for routine histopathological analysis. Statistically significant differences were determined by using Mann–Whitney U test.

### 4.13. Statistics

All statistical analyses were done using Prism 5.0 (GraphPad Software Inc., La Jolla, San Jose, CA, USA). Statistically significant differences were then determined using two-tailed Student’s *t*-test, Mann–Whitney U test, or Kruskal–Wallis test where appropriate (as indicated in the text and figure legends). Data are expressed as the means ± standard errors (SEs). Statistical significance was considered at a minimum value of *p* < 0.05.

## 5. Conclusions

Our study elucidates the regulatory mechanism of KRAS/RhoA/Wnt in NSCLC metastasis driven by KRAS^G12V^ and KRAS^G12D^. We demonstrate that RhoA act as an upstream regulator in modulating the activation of Wnt/β-catenin pathway. The KRAS^G12D^ mutation activate RhoA and further abolish the activation of Wnt/β-catenin signaling. In contrast, KRAS^G12V^ mutation inactivate RhoA and further provoke Wnt/β-catenin signaling. Moreover, the higher ability of migration induce by KRAS^G12V^ mutation may also inhibit by Wnt inhibitors. We prove that the KRAS mutations are different even with a single nucleotide substitution. Our data provide important insights and evidences of KRAS^G12V^ induced metastasis may promote by Wnt/β-catenin signaling. The disease induced by KRAS mutations are not one disease.

## Supplementary Materials

The following are available online at https://www.mdpi.com/2072-6694/12/4/837/s1, Figure S1: The mutation status of KRAS in H838 KRAS isogenic cells, Figure S2: The gene ontology and pathway analysis in H838 KRAS isogenic cells, Figure S3: The MAPK pathway in H838 *KRAS* isogenic cells, Figure S4: GEF-H1 was increased in H838 *KRAS^G12D^* cells. Figure S5: KRAS G12D inactivate the activity of WNT/β-catenin in MRC-5 cells. Figure S6: The RhoA suppress the expression of β-catenin in H838 KRAS isogenic cells. Figure S7: The mutation status of KRAS in KRAS-mutant NSCLC cells. Figure S8: The migration ability of *KRAS ^G12V^*mutant NSCLC cells were attenuated by LGK974, Table S1: Quantitative real-time PCR primers used in this study, Table S2: List of antibodies used in western blotting, Figure S9: The uncropped blots and molecular weight markers of Figure 5, Figure S10: The uncropped blots and molecular weight markers of Figure 6.
