# Supplementary material for: The Inhibition of Wnt Restrain KRASG12V-Driven Metastasis in Non-Small-Cell Lung Cancer
Source: Cancers (Basel). 2020 Mar 31;12(4):837. doi: 10.3390/cancers12040837 (PMC7226522; doi:10.3390/cancers12040837)
Supplement: Supplementary file 1 [file cancers-12-00837-s001.pdf]

Supplementary Materials: The Inhibition of Wnt Restrain *KRAS*^{G12V}-Driven Metastasis in Non-Small-Cell Lung Cancer

Pei-Shan Hung, Ming-Hung Huang, Yuan-Yeh Kuo and James Chih-Hsin Yang

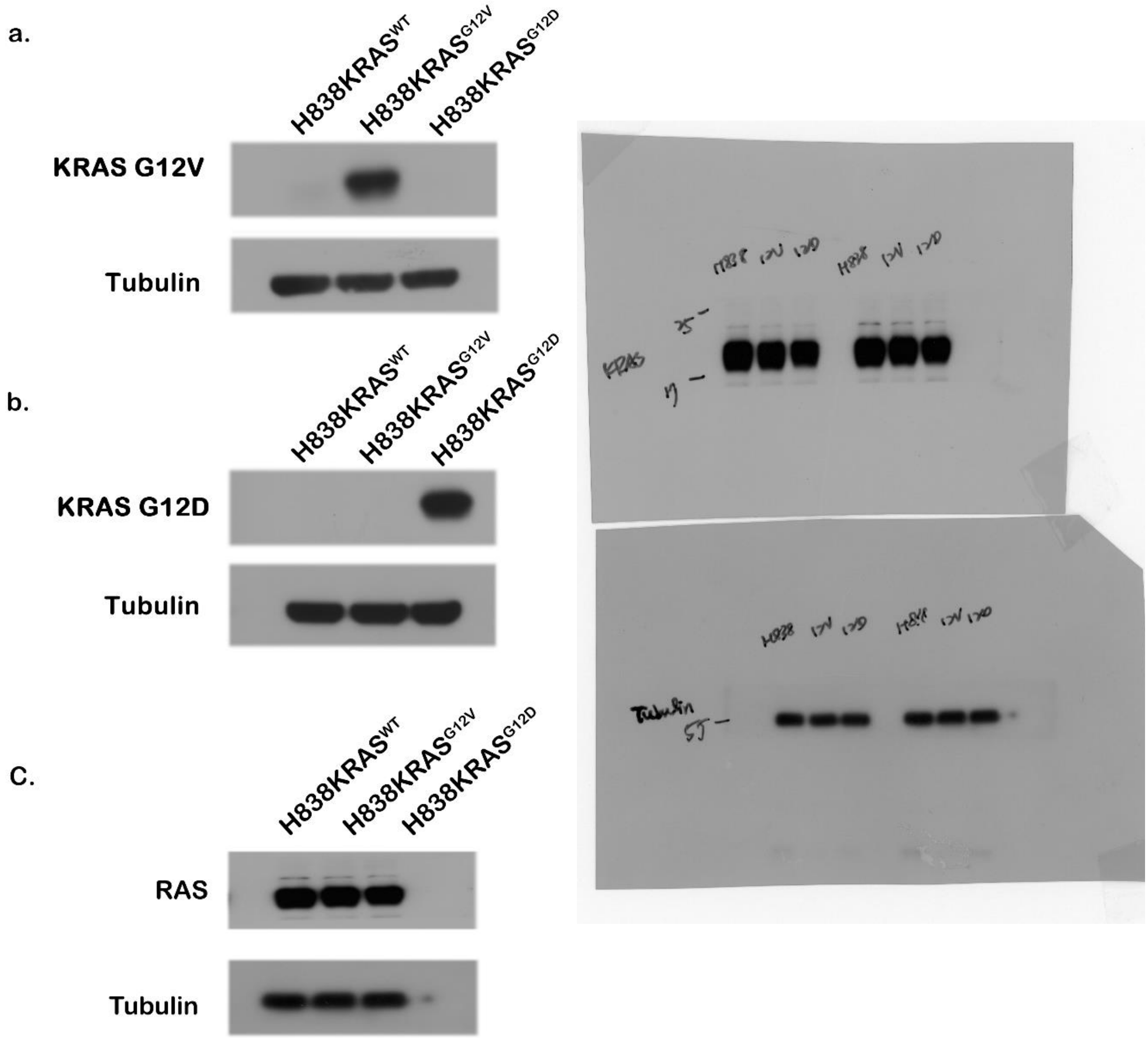

Figure S1. The mutation status of KRAS in H838 KRAS isogenic cells. (**a**). The expression of KRAS ^{G12V} in H838KRAS ^{G12V} cells. (**b**)The expression of KRAS^{G12D} in H838KRAS ^{G12D} cells. (**c**)The western blotting of RAS expression in H838 isogenic cells.

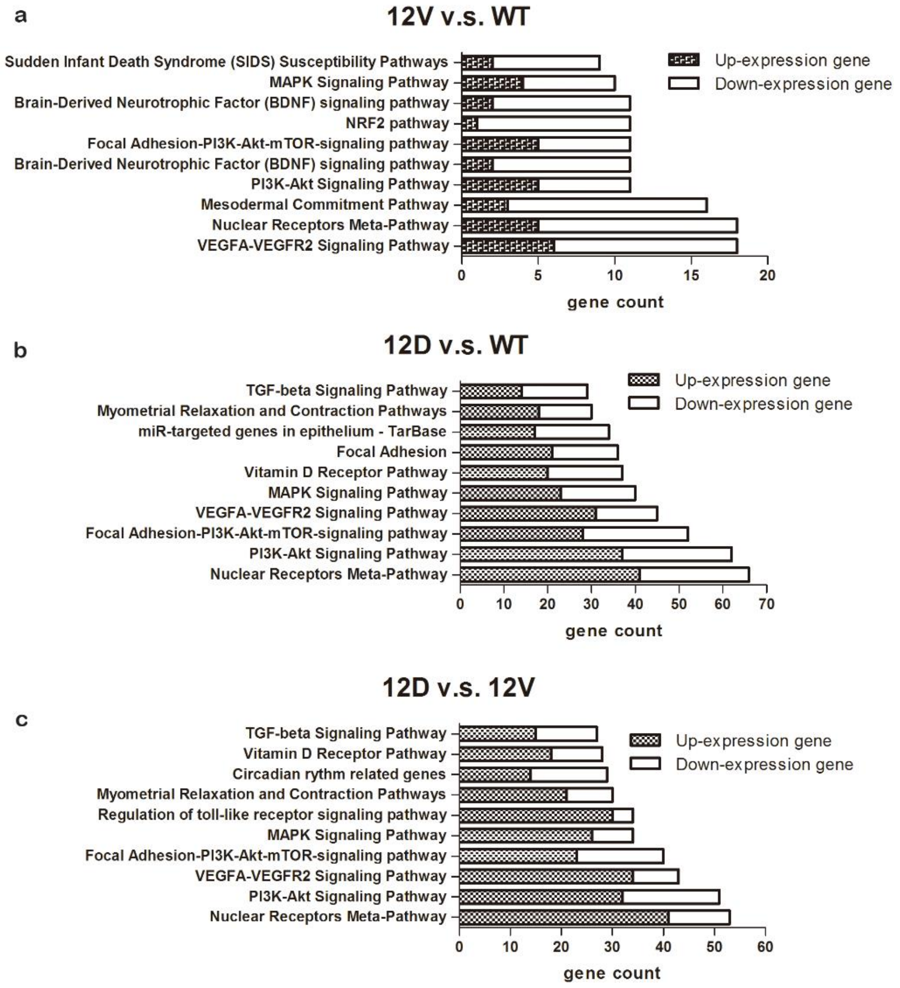

Figure S2. The gene ontology and pathway analysis in H838 KRAS isogenic cells. The top 10 Pathways were predicted to be associated with H838KRAS^{WT}, H838KRAS ^{G12V}, and H838KRAS^{G12D} on *Y* -axis and genes count on *X* -axis.

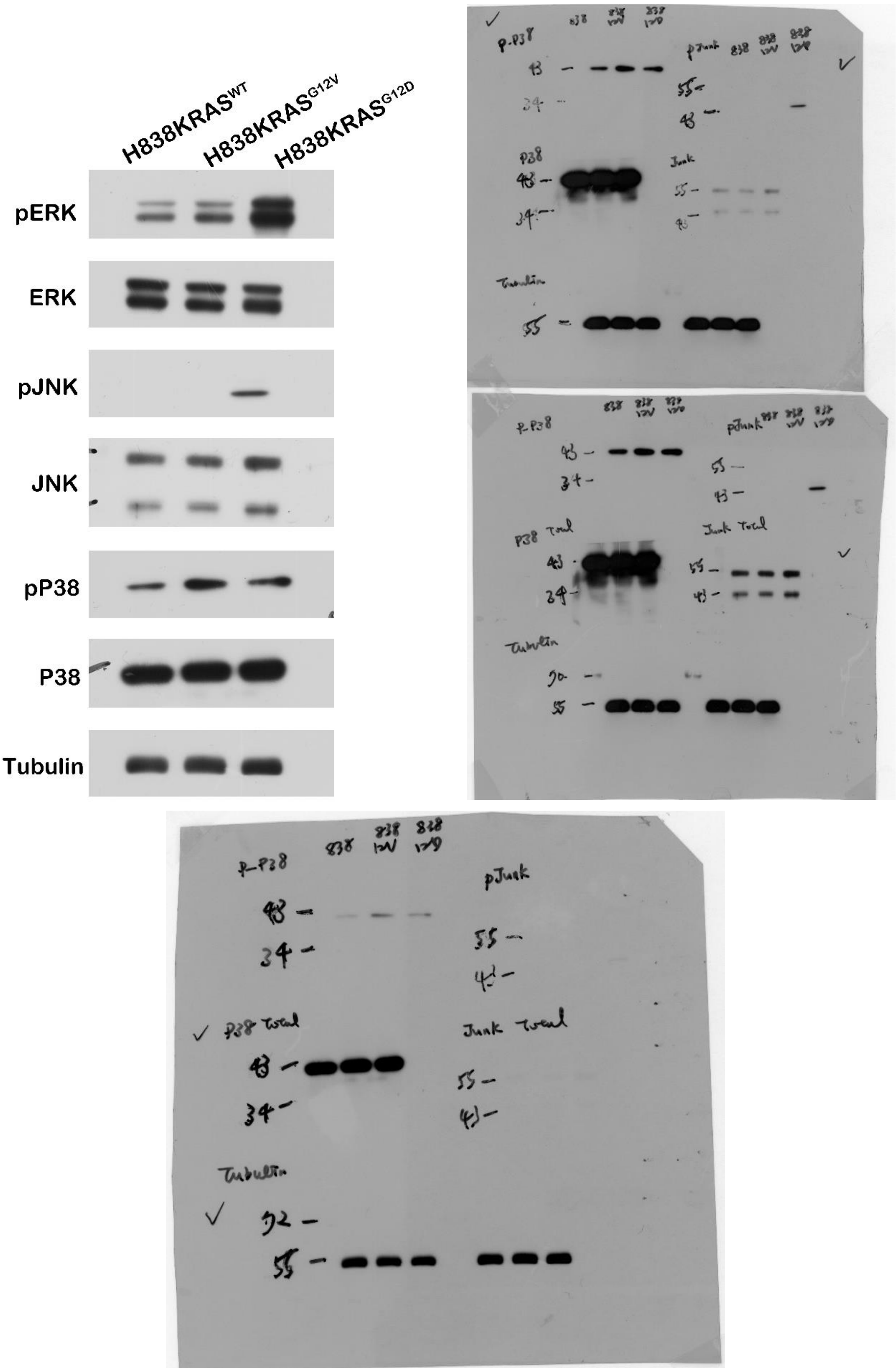

Figure S3. The MAPK pathway in H838 KRAS isogenic cells. Immunoblot blot were probed with ERK (phospho/Total), JNK (phospho/Total) and p38 (phospho/Total) antibody. Tublin was used as an internal control.

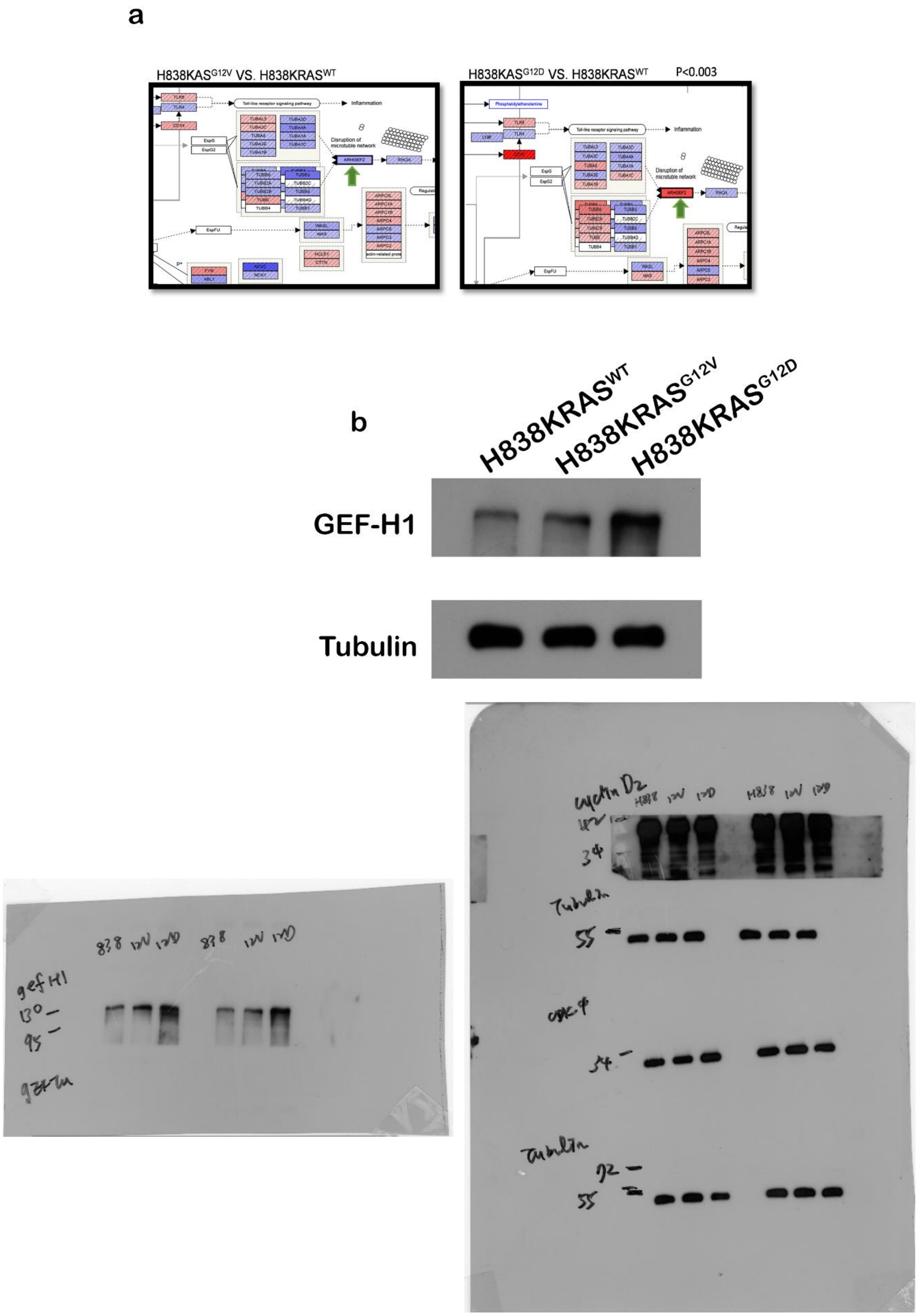

Figure S4. GEF-H1 was increased in H838KRAS^{G12D} cells. (**a**). The analysis of Transcriptome Analysis Console (TAC) of GEF-H1 and GEF-H1 related pathway. (**b**). The immunoblot analysis of GEF-H1 in H838KRAS isogenic cells. Tubulin was used as an internal control.

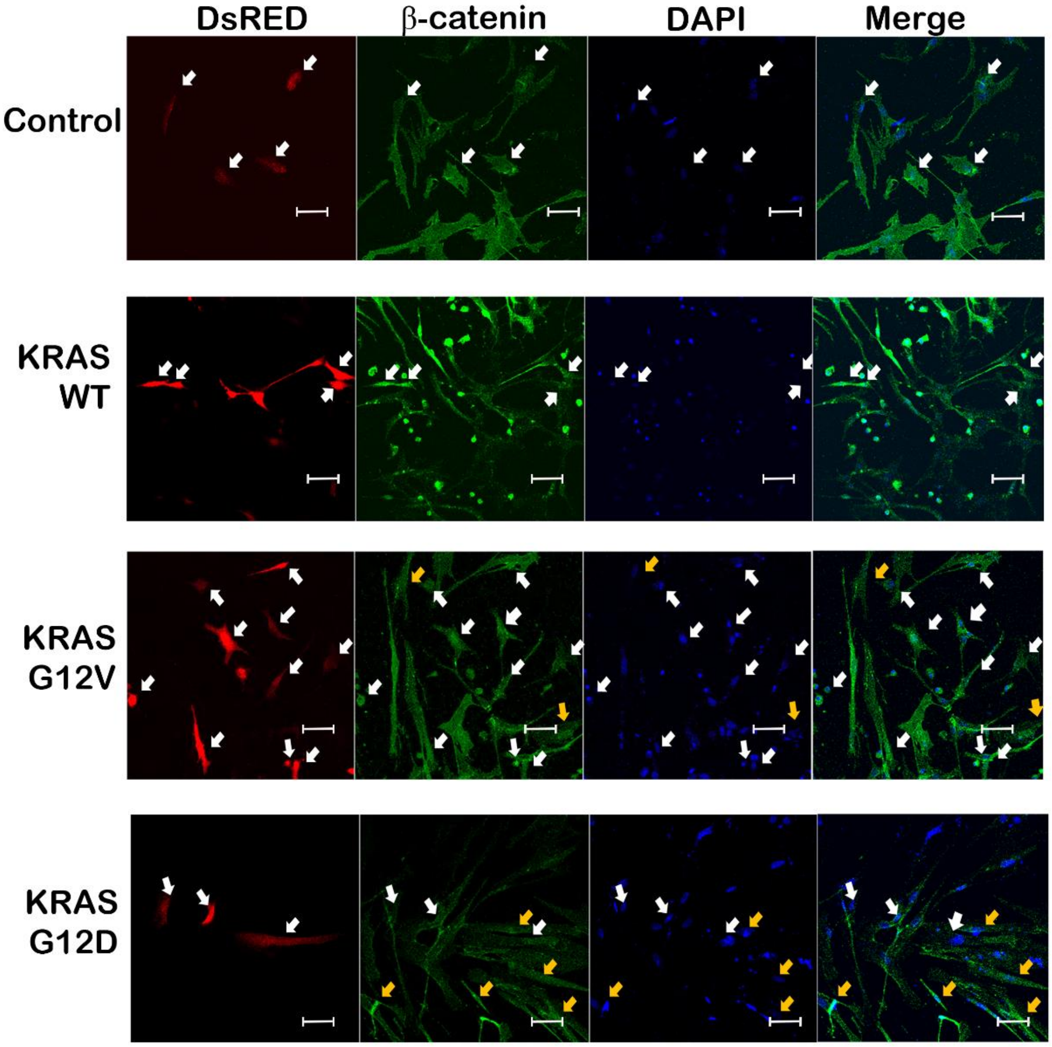

Figure S5. KRAS G12D inactivate the activity of WNT /β-catenin in MRC-5 cells. KRAS G12D did not induce WNT /β-catenin translocation into nucleus. White arrows indicate the plasmid transfected cells. Yellow arrows indicate the non-transfected cells. MRC-5 cells were transfected with DsRed-tagged KRAS WT, KRAS G12V, and KRAS G12D plasmid. IF staining after EGF stimulation. β-catenin is visualized with green fluorescent. Hochest3342 is the blue nuclear stain. Immunoreactivity was captured by confocal microscopy. Scale bar 50 μm.

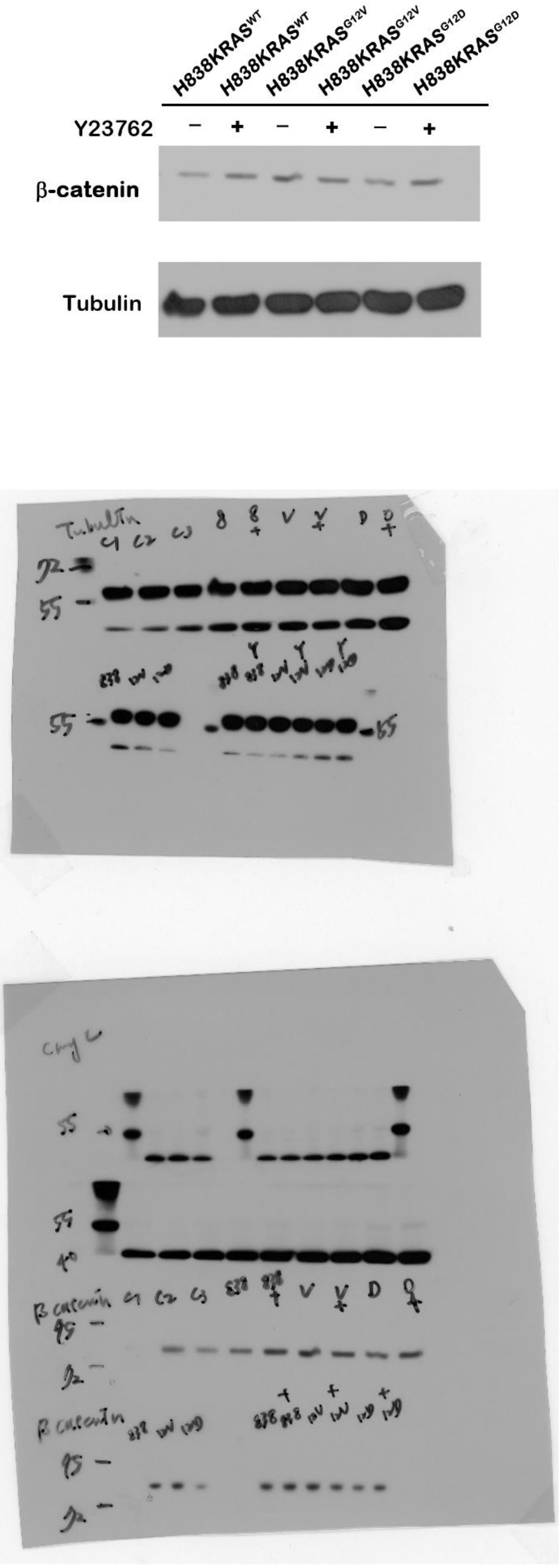

Figure S6. The RhoA suppress the expression of β-catenin in H838 KRAS isogenic cells. The western blotting of β-catenin in H838 KRAS isogenic cells after 30μM Y23762 treatment (RhoA inhibitor).

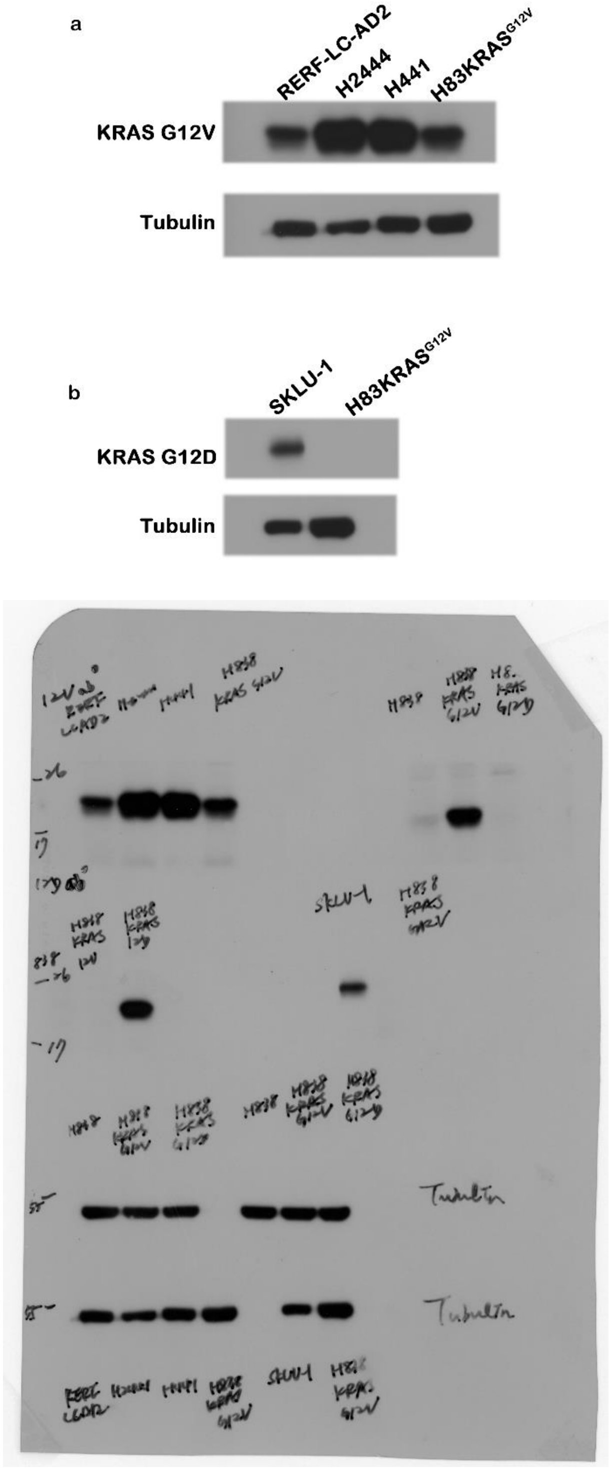

Figure S7. The mutation status of KRAS in KRAS-mutant NSCLC cells. (**a**) The expression of KRAS ^{G12V} in RERF-LC-AD2, H441, H2444, and H838KRAS ^{G12V} cells. H838 KRAS ^{G12V} cells as a positive control. (**b**) The expression of KRAS^{G12D} in SKLU-1 and H838KRAS^{G12V} cells. H838KRAS^{G12V} cells as a negative control.

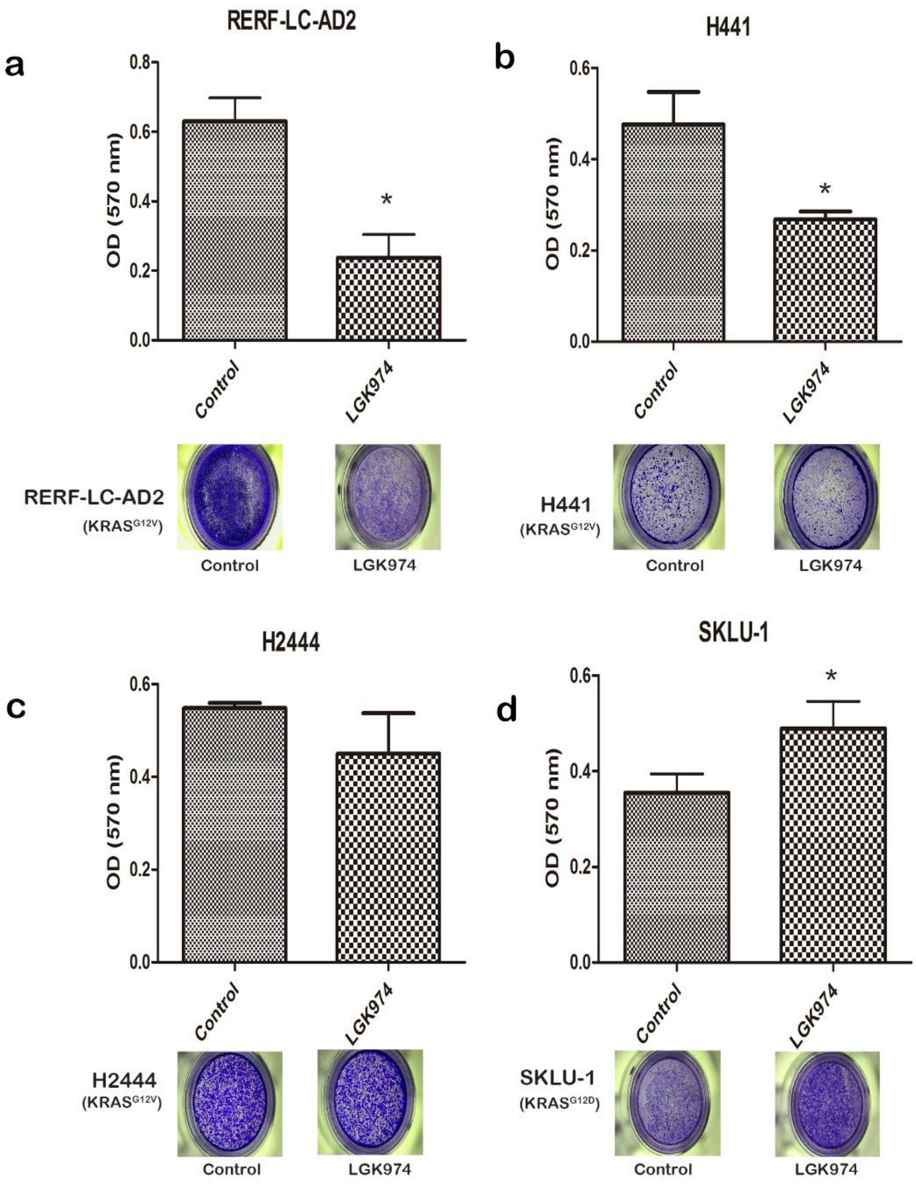

Figure S8. The migration ability of KRAS^{G12V}mutant NSCLC cells were attenuated by LGK974. (**a**). The migration assay of RERF-LC-DA2 after 20nM LGK974 treatment. (**b**). The migration assay of H441 after 20nM LGK974 treatment. (**c**). The migration assay of H2444 after 20nM LGK974 treatment. (**d**). The migration assay of SK-LU-1 after 20nM LGK974 treatment. The values represent the means ± s.d. of three independent assays ($n = 3$, $*p < 0.05$)

Table S1. Quantitative real-time PCR primers used in this study.

Gene	Product Number
CDH1	Hs01023894_m1
CDH2	Hs00983056_m1
SNAI1	Hs00195591_m1
SNAI2	Hs00161904_m1
VIM	Hs00958111_m1
CTNNB1	Hs00355049_m1
GAPDH	Hs99999905_m1

Table 2. List of antibodies used in western blotting.

Antibody	Name Company

β-catenin (#8480)	Cell Signaling
Vimentin (#5741)	Cell Signaling
Snail (#3879)	Cell Signaling
Slug (#9585)	Cell Signaling
Cyclin D1(GTX108624)	GeneTex
c-Myc (GTX103436)	GeneTex
Tubulin (ab12546)	Abcam
N-cadherin (ab76011)	Abcam
E-cadherin (ab40772)	Abcam
Ras (#3965)	Cell Signaling
Ras G12V mutant (#14412)	Cell Signaling
Ras G12D mutant (#14429)	Cell Signaling
pP38 (#9211)	Cell Signaling
P38 (#8690)	Cell Signaling
pJunk (#9251)	Cell Signaling
Junk (#9252)	Cell Signaling
pERK (#9101)	Cell Signaling
ERK (#9102)	Cell Signaling

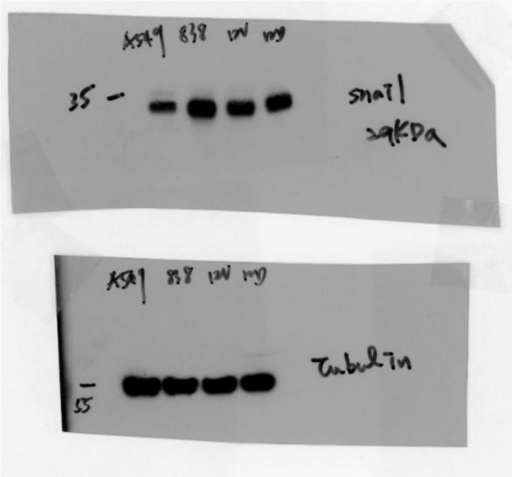
A549 838 12V 12D
35
snail
29KDa
A549 838 12V 12D
Tubulin
55

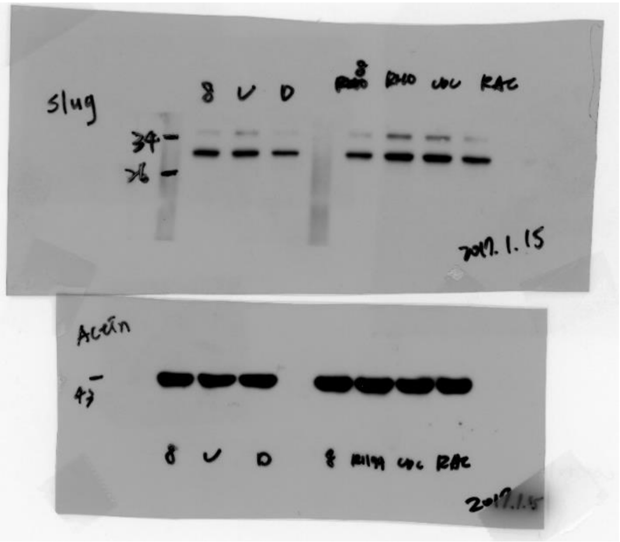
slug
34
26
2017.1.15
Actin
43

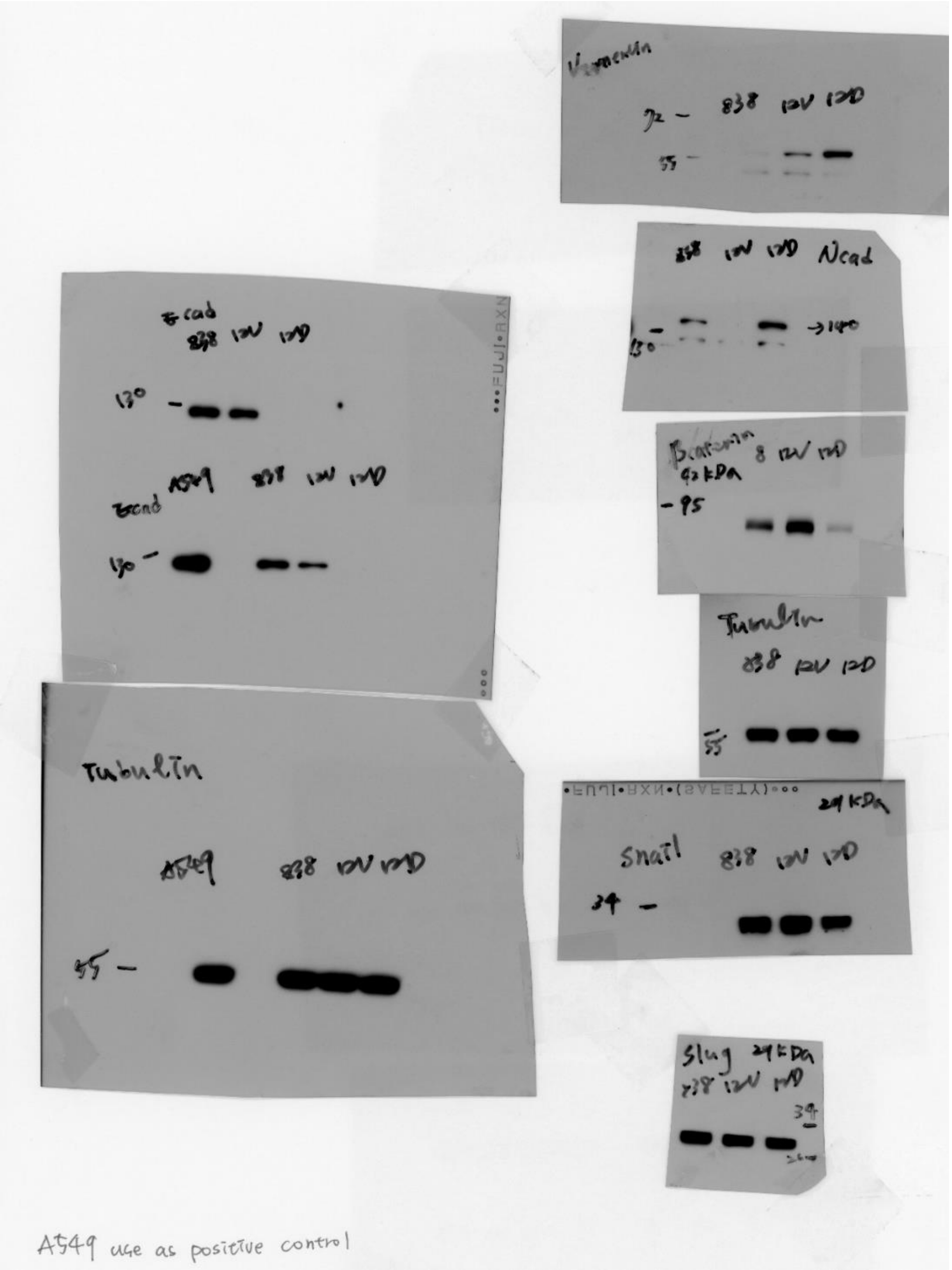
Vimentin
838 12V 12D
55
838 12V 12D Ncad
140
130
Ecad
838 12V 12D
130
A549 838 12V 12D
130
Tubulin
838 12V 12D
55
Tubulin
A549 838 12V 12D
55
29 KDa
snail 838 12V 12D
34
slug 29KDa
838 12V 12D
34
A549 use as positive control

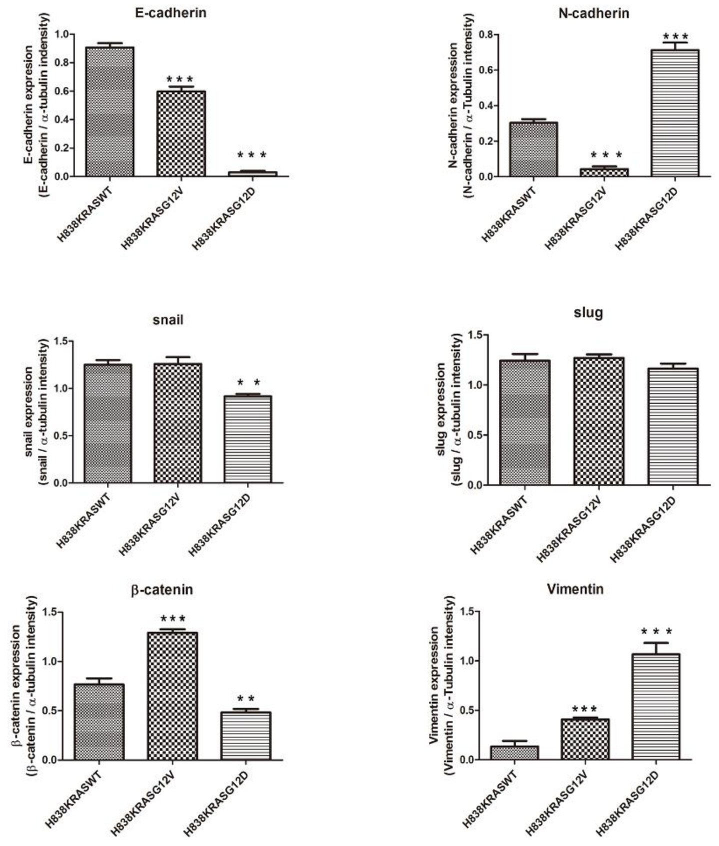

Figure S9. The uncropped blots and molecular weight markers of Figure 5.

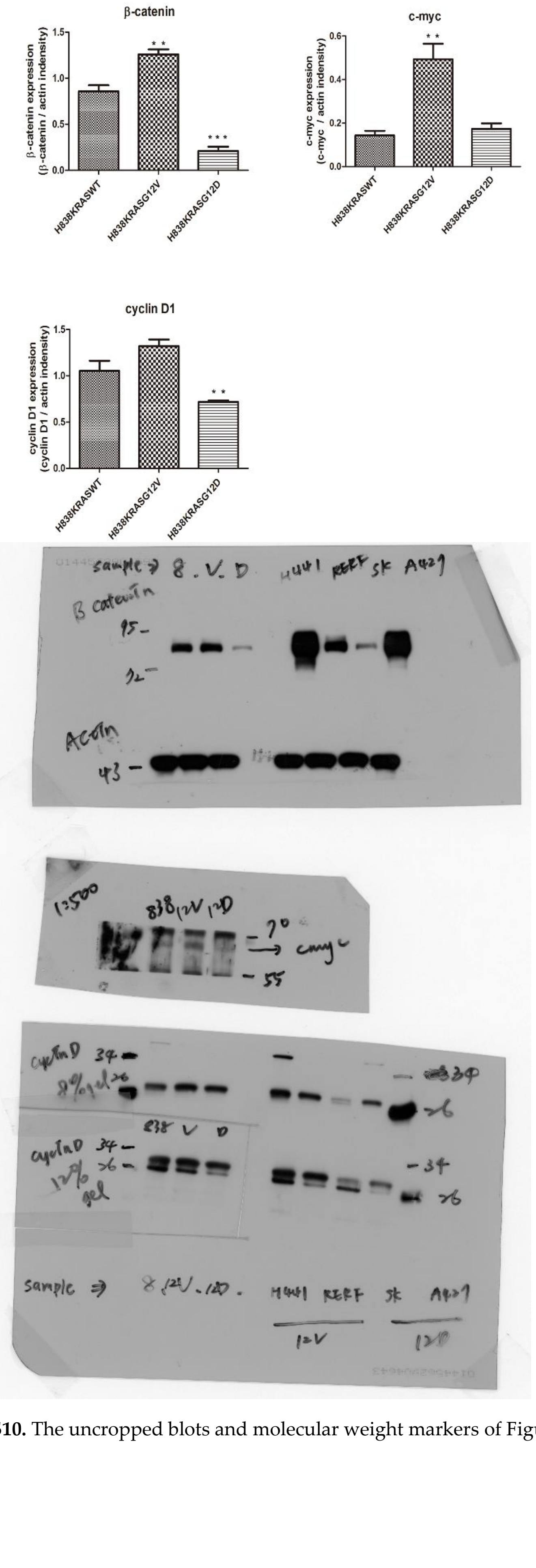

Figure S10. The uncropped blots and molecular weight markers of Figure 6.

© 2020 by the authors. Licensee MDPI, Basel, Switzerland. This article is an open access article distributed under the terms and conditions of the Creative Commons Attribution (CC BY) license (http://creativecommons.org/licenses/by/4.0/).
